# Tobacco spending and the perceived cost of tobacco among smokers living with HIV and receiving treatment at outpatient clinics in Viet Nam: A mixed methods study

**DOI:** 10.1371/journal.pone.0327490

**Published:** 2025-07-02

**Authors:** Gloria Guevara Alvarez, Thanh Hoang, Reet Kapur, Claire Nguyen, Mari Armstrong-Hough, Trang Nguyen, Nam Nguyen, Hoang Van Minh, Donna Shelley

**Affiliations:** 1 Department of Health Policy and Management, New York University, School of Global Public Health, New York, New York; 2 School of Public Health and Community Medicine, University of Goethenburg, Göteborg, Sweden; 3 Institute of Social and Medical Studies, Ha Noi, Viet Nam; 4 Ha Noi University of Public Health, Ha Noi, Viet Nam; Hue University of Medicine and Pharmacy, VIET NAM

## Abstract

**Background:**

Tobacco use among people living with HIV (PWH) is 2–3 times higher than among HIV-negative individuals. In Viet Nam, over 50% of men living with HIV use tobacco. Reducing smoking is important to improving disparities among PWH who smoke including their higher exposure to chronic disease. However, data on tobacco spending as well as the potential impact of tobacco policies, such as taxation, is limited among PWH. Viet Nam has one of the lowest taxes on tobacco in the world, thus underutilizing this tool. Our study aims to understand tobacco spending among PWH who smoke as well as examine the financial burden of tobacco use, and their perceptions about the affordability of tobacco products.

**Methods:**

We conducted qualitative interviews (n = 24) that explored smoking behavior and perceptions about the cost of tobacco, alongside cross-sectional quantitative surveys (n = 75) that assessed daily tobacco expenditures and the proportion of income spent on tobacco. We applied convergent parallel design to these two independent samples taken from the same study population of PWH enrolled in a tobacco use treatment study at HIV clinics in Ha Noi, Viet Nam.

**Results:**

Participants spent 7.47% of their annual income on tobacco products. Tobacco dependence was positively associated with higher daily expenditure on tobacco. Qualitatively, participants did not describe tobacco-related spending as a financial burden and did not consider the price of cigarettes as a motivation to quit. However, participants acknowledged that smoking is still a costly expenditure and indicated that quitting would yield financial savings which could be redirected to other household needs.

**Conclusion:**

Despite spending a considerable percentage of their income spent on tobacco, most participants perceived cigarettes as affordable. This may reflect our sample’s strong socioeconomic resources as well as the low price of cigarettes in Viet Nam, which may be too low to cause financial hardship and thus reduce motivation to quitting.

## Introduction

Smoking prevalence among people living with HIV (PWH) is two to three times higher than that of the general population [1]. For example, in the United States, 40% of PWH smoke compared to about 20% of tobacco smokers in general population [2]. This pattern is consistent across world regions and countries with different income levels. Moreover, smoking is mostly concentrated among men [3–7]. The estimated proportion of PWH on antiretroviral therapy (ART) who were active smokers in Eastern and Southern Africa is close to 35.9% and in Asia and the Pacific, close to 25.9%. Crucially, tobacco use among PWH substantially increases the risk of morbidity and mortality compared with nonsmoking HIV counterparts, threatening gains in long-term survival for PWH [8–11]. To reduce these disparities, it is important to understand the role that existing tobacco control interventions (e.g., tobacco use treatment) and policies (e.g., tobacco taxation) play in promoting cessation in this medically vulnerable population.

Taxing tobacco products is one of the most effective policies for reducing tobacco use [12]. In the United States, increased cigarette taxes are associated with sustained quitting among the general population [13]. This positive impact of extends to LMICs, where more than 84% of tobacco users live. The Gambia saw a 60% reduction in cigarette importation after implementing a steady increase in taxation from 2012 to 2018, and similarly, Colombia experienced a 34% reduction in cigarette consumption in 2019 following a 300% increase in cigarette taxes from 2016–2018 [14]. The reason for the efficacy of these policies is that higher prices of tobacco create a financial burden, thereby deterring tobacco purchases and leading to decreased tobacco use [15].

However, this desired outcome may not always be achieved. Studies among the general population suggest that spending on tobacco products redirects resources away from purchasing items like food, paying for housing and household bills, which contributes to overall financial difficulties in LMICs [16]. In Asian and Southeast Asian countries like Cambodia, China and Indonesia, where smoking rates are the highest in the world, the percentage of total annual income spent on tobacco ranges from 3.6% to 7% [17–21]. This tobacco-related expenditure “crowds out” spending required for on essential needs, such as education, food, and housing particularly in low- and middle-income households [22]. However, these metrics pertain to the general population, and there is a lack of data on the effectiveness of tobacco tax policies specific to PWH who smoke. While tobacco taxation is an essential component of a comprehensive tobacco control strategy, it is also unclear how high-risk subpopulations like PWH are impacted by these policies and if additional measures are needed to deter smoking behavior.

In Viet Nam, among the general population, it is estimated that the annual average tobacco spending was 2.7 million VND in 2020 and 1.9 million VND in 2016 [23,24]. However, the rate of tobacco expenditure among PWH is not known, even though it is estimated that close to 60% of PWH smoke, which presents a significant disease burden. Viet Nam has one of the lowest taxes on tobacco in the world [25]. Its cigarette taxation is largely based on the ad valorem tax structure, which results in taxes constituting 36.7% of the retail price that consumers pay for cigarettes [26]. Additionally, Viet Nam does not levy a tax on tobacco used for waterpipes. Therefore, while this taxation percentage of almost 40% may seem substantial, the country still scores low (0.75 out of 5.0) on the Tobacconomics Cigarette Tax Scorecard. This scorecard measures the effectiveness of cigarette tax policies based on several factors like price, affordability, tax structure, and the proportion of tax present in the retail price of cigarettes, and ranks countries on a 5-point scale where 0 indicates lowest policy effectiveness and 5 indicates highest policy effectiveness [27]. To explore why high rates of smoking persist among PWH in Viet Nam despite existing tobacco taxes, this study will examine the financial burden of tobacco use among PWH smokers in Viet Nam to gain insights into the effectiveness of current tobacco tax policies and offer policy recommendations. The financial burden is examined both quantitatively, using tobacco spending data, and qualitatively, using interviews with patients on the perceived cost of tobacco.

This study is among the first to focus on the financial burden of tobacco use among PWH smokers in Viet Nam or any low- and middle-income country (LMIC). While data from high-income countries (HICs) show that tobacco use and expenditure can exacerbate financial hardship among PWH, comparable evidence is still lacking in LMICs. Our study aims to address this gap by assessing if this relationship persists in an LMIC, such as Viet Nam, and discuss the impact of cigarette policies targeting reduced spending, such as increased taxation policies. Low tobacco taxation conditions, such as the low price of cigarettes, may facilitate tobacco use and undermine efforts to reduce tobacco consumption [28]. By exploring how tobacco spending affects PWH in this setting, our findings can inform the design of taxation policies that effectively reduce smoking and its financial burden among vulnerable populations.

## Materials and methods

### Study design

We employed a mixed methods approach, convergent parallel design [29,30], to collected qualitative and quantitative data, which provide distinct but complementary data on tobacco cost, allowing us to obtain a complete understanding of tobacco affordability. We used two independent samples, a quantitative sample and a qualitative sample, which were made up of PWH who smoke and were receiving care at OPCs in Ha Noi. These patients were enrolled in a larger randomized controlled trial (RCT), VQuit, that evaluated the effectiveness of smoking cessation interventions among PWH [31].

#### Study samples.

**Fig 1:**
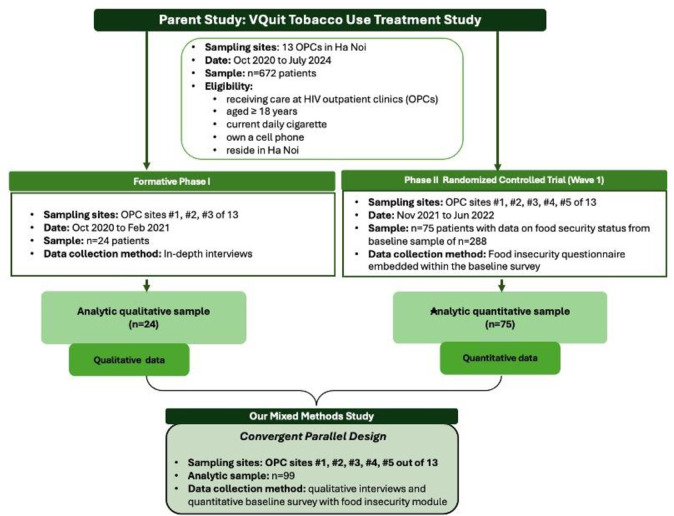
Study flow chart of sampling and timeline. Note Note: The flow of participant samples and data sources used in the present mixed methods study were nested within the VQuit parent study. The analytic sample includes 24 qualitative interviews from the formative phase of the study and quantitative survey responses from 75 patients who completed the food security questionnaire within the baseline survey from the RCT phase of the study.

The participants in the qualitative sample included patients who only participated in the formative phase of the main VQuit study (October 3, 2020 to February 10, 2021), and were enrolled in one of three OPCs (Fig 1). The qualitative sample size (n = 24) was determined based on the number of interviews needed to reach saturation of the formative themes focused on adapting tobacco use treatment to HIV care, which was defined as the point at which any additional interviews yielded no new themes or variations [32]. The quantitative sample was comprised of participants who completed baseline surveys at the time of their enrollment in the main VQuit RCT (November 15, 2021 to June 17, 2022). The baseline survey assessed tobacco use behaviors, other substance use, food security status and daily tobacco expenditures. Survey data were collected electronically by a Research Assistant (RA) at the time of study enrollment using REDCap software. The quantitative sample, n = 75, was determined by constraints on time and budget.

#### Eligibility to participate in the VQuit study.

Participants were eligible if they were receiving care at OPCs included in the VQuit study, were aged ≥ 18 years, were current daily cigarette and/or waterpipe smokers, owned a mobile phone and lived in Ha Noi. For the qualitative sample, eligibility was determined if they were enrolled in the formative phase; for the quantitative sample, if they were enrolled in the RCT.

#### Recruitment and enrollment in VQuit study.

Trained staff screened participants for current tobacco use consecutively as they registered for their visits at the OPC. Participants who reported using tobacco and were willing to learn about the study were introduced to the onsite RA who confirmed eligibility. The formative phase of the VQuit study has been reported in detail elsewhere. In brief, this formative phase focused on collecting data from providers and patients at participating OPCs through qualitative interviews, to guide the adaptation of intervention components. These adaptations were pilot-tested prior to initiating the RCT [31]. The recruitment and enrolment process was the same for both the qualitative and quantitative samples, with the exception that we obtained verbal consent for the qualitative interviews and written consent from participants who were enrolled in the RCT and responded to the quantitative survey [31].

### Measures

Participants from the main RCT were included in this analysis if they responded “yes” that they currently smoke cigarettes somedays or every day. To assess if participants were waterpipe users (in addition to cigarette users) we used a similar yes/no question. Dual users were those participants who responded yes to using both products (cigarettes and waterpipe).

Tobacco spending was assessed using two questions that collected responses as continuous variables. First, participants were asked, *“How much did you spend the last time you purchased tobacco?”* (e.g., 50,000 VND); and second, *“How many days did that purchase last?”* (e.g., 10 days). From these data we calculated three tobacco spending variables: **1) Daily spending on cigarettes**; which was defined as the total amount participants reported spending on only cigarettes (VND) divided by the number of days between this purchase and the next purchase; **2) Daily spending on tobacco (cigarettes and waterpipe)**; which was defined as the total amount participants reported spending on each of these products the last time they were purchased divided by the number of days between each purchase and; **3) Percent of annual income spent on tobacco**; which was defined as the total amount spent on tobacco per year (daily spending on tobacco multiplied by 365 days) divided by annual income.

Participants’ readiness to quit smoking was categorized as: (1) not interested in quitting, (2) planning to quit in the next 6 months (3) planning to quit in the next 30 days, and (4) currently trying to quit [33]. How many cigarettes smoked per day was recorded as a continuous variable. Tobacco dependence was assessed using the 10-point Fagerstrom scale [34].

Sociodemographic characteristics included age, household size, gender, living arrangement, marital status, education, and employment status. Food security status was assessed using the validated Household Food Insecurity Access Scale (HFIAS), where participants were classified into two groups, “mild-to-little food insecurity” and “moderate-to-severe food insecurity” [35]. The HFIAS has been validated among PWH and has been reported to have high reliability in LMICs, including Viet Nam, although it has not been validated in Viet Nam [36–39]. To collect data on substance use, we measured the degree of alcohol consumption using the AUDIT-C [40]. Participants also self-reported illicit drug use, including lifetime use (yes/no) and recent use within the past 3 months (yes/no), and current enrollment in methadone treatment (yes/no).

We transformed and dichotomized two variables: (1) the four-category readiness to quit variable; to compare those who were trying to quit (1 – trying to quit) with those who were not planning to quit, were quitting in the next 30 days, or quitting in the next 6 months (2 – not trying to quit); and (2) the five-category Fagerstrom scale; to compare those who had very high and high tobacco dependence (1 – addicted) versus those who had medium, low and very low dependence (2 – not addicted).

### Analysis

We analyzed quantitative and qualitative data separately and then merged the findings for joint interpretation [30]. This approach gives equal weight or priority to quantitative and qualitative data, ensuring that each data method exerted equal influence in informing our interpretation, thereby strengthening the triangulation and validity of our findings. Each strand was first analyzed independently, after which the results were merged and compared during the overall interpretation. Specifically, we first analyzed the results on tobacco spending (from the quantitative sample) and tobacco affordability (from the qualitative sample). We then examined these two results to see how they relate to each other, and finally, drew conclusions to reflect on the insights collected from their comparison. For example, we identified to what extent and in what ways the two sets of results converge, diverge or relate to each other, and/or how they can be combined to create a more comprehensive understanding of tobacco-related spending and financial burdens.

To analyze the quantitative data, we generated descriptive statistics and conducted bivariate analysis with a significance level of 0.05. We then developed generalized linear regression models to assess the effect of our dependent variable, tobacco spending, on selected covariates. We staggered in covariates and confounders, identified from the literature (age, income), and those that were statistically significant in bivariate analysis (years living with HIV, lives alone). Therefore, the independent variables used in the regression model were age, years living with HIV, living alone and income. We conducted robustness checks by running linear models that were robust to the violations of normality and by running male-only models of both generalized and linear models, given the expected small number of women in our study sample due to the low number of female smokers in Viet Nam.

For the qualitative data, two authors who are bilingual native speakers of Vietnamese with experience in qualitative methods (TH, CN), used a directed content analysis method to analyze interview transcripts from participants [41,42]. This approach starts with using theory, in this case the Consolidated Framework for Implementation Research (CFIR), Theoretical Domains Framework and the socio-ecological model (SEM) [43–46], to guide coding, and subsequently adding codes as needed to reflect the themes in the data. These interviews were conducted in-person, transcribed in Vietnamese, and the transcriptions were translated into English, which were then analyzed.

In the first stage, using a deductive approach, TH and CN identified potential codes and themes related to the cost of tobacco and its financial impact from a codebook that was developed during a previous analysis on the same qualitative sample [47]. Subsequently, they reviewed audio recordings and interview transcripts in Vietnamese to identify and highlight units or text excerpts that referred to tobacco purchases and participant-perceived financial effects of tobacco smoking. Next, all the highlighted excerpts were coded using the predetermined codes from the previously developed codebook. Texts that did not fit into the codebook would receive a new code. All coding was conducted in Atlas.ti software [48].

To synthesize the quantitative and qualitative components, we adopted the Convergent Parallel Analysis method described by Creswell [30]. We selected key findings from the quantitative data and used the qualitative data to understand how tobacco costs are perceived by smokers and how these costs may contribute to the financial burden of tobacco use. The quantitative and the qualitative data offered a deeper understanding of perceptions about the economic impact of tobacco use, as well as the attitudes surrounding tobacco use and perceived financial strain it causes.

The study was approved by the New York University (no. il9–01783) and Institute of Social and Medical Studies (no. 00007993) Institutional Review Boards.

## Results

We present herein results from each of the two samples, highlighting their demographic similarity particularly in terms of age and income.

### Surveys

#### Demographic characteristics of survey participants.

The majority of participants were men (92.0%) having a mean (SD) age of 43.2 (5.75) years. Approximately half of them were married (45.3%). The mean (SD) household size was 2.71 (0.653) people, and 89% of participants lived with family compared to those who were single. Participants had a mean (SD) annual income of 186 million (150 million) VND or 7,666 (6,122) USD, with a range of [36,000,000; 1,200,000,000]. The mean (SD) percent of income spent on food per year was 12.3 (15.6), and 12% of participants experienced food insecurity. Almost the entire sample (97.3%) reported attaining secondary or higher educational levels. The number of participants who were undergoing methadone treatment was 18 (24.0%) The rate of unemployment was 2.7% (Table 1).

**Table 1:** Demographic and tobacco use characteristics of HIV+ smokers reporting on food security status in Viet Nam at baseline (n = 75).

Sociodemographic variable	Total (n = 75)*Mean (SD)* or Number (%)
Gender	
Male	69 (92.0)
Female	6 (8.0)
Age	*43.2 (5.75)*
Household size	*2.71 (0.653)*
Lives with family (vs single)	67 (89.0)
Married status	
Married	34 (45.3)
Single/never married/divorced	41 (54.7)
Annual income (VND)	*186 million (150 million)*
Food insecure	9 (12.0)
% of income spent on food per year (n = 58) smokers) )	*12.3 (15.6)*
Education	
Primary school (Grade 1–5) or fewer years	2 (2.7)
Secondary school (Grade 6–9)	23 (30.7)
High school (Grade 10–12)	27 (36.0)
Vocational training/College	13 (17.3)
University	10 (13.3)
Methadone treatment	18 (24.0)
Unemployed	2 (2.7)

#### Tobacco use and spending characteristics.

The majority of participants (n = 60, 80%) were dual users. The mean (SD) of cigarettes smoked per day was 16.6 (8.0). Annually, the mean (SD) percent of income spent on tobacco per year was 7.47 (7.11) with the median and range being 4.65 [0.456, 34.8]. The mean (SD) daily cost of cigarettes was 25,000 (12,100) VND or 1.26 (0.51) USD (median = 30,000 [3000 to 60,000] VND); for dual users, the mean (SD) daily cost of waterpipe use was 2,030 (1,400) VND or 0.08 (0.06) USD (median = 1,670 [333 to 5,000] VND). The mean (SD) daily amount spent on tobacco products by dual users was 26,000 (12,300) VND or 1.07 (0.50) USD. Almost half of the participants (n = 40, 53.3%) reported high or very high tobacco dependence and almost the same proportion (n = 35, 46.6%) were currently trying to quit, which was the only active option within the readiness to quit smoking measure used. In terms of substance use characteristics, lifetime illicit drug use was reported by 88% (n = 66) of participants, with 30.7% (n = 23) reporting recent use in the last three months. The mean (SD) alcohol consumption score was 4.32 (3.46) (Table 2).

**Table 2:** Participant characteristics by tobacco dependence.

		Tobacco spending Mean (SD) or n(%)					Tobacco use characteristics Mean (SD) or n(%)		Readiness to quit n (SD) or n(%)	Substance use Mean (SD) or n(%)		
	Annual Income	% of income spent on tobacco per year (VND)	Daily amount spent on tobacco (VND)	Daily amount spent on cigarettes (VND)	Daily amount spent on waterpipe (VND) (n = 37)	Total spent on tobacco per year	Dual users	Cigarettes smoked per day∞	Readiness to quit	Drug use in last 3 months	Drug use ever	Alcohol use
**Tobacco dependence level**												
Not addicted: Very low/low/ medium tobacco dependence n = 38	215m (197m)	4.11 (2.44)	19,600 (9,550)	18,900 (9,590)	1,580 (1,110)	7,154,000 (3,335,003)	22 (78.6)	12.57 (6.39)	16 (45.7)	9 (25.7)	29 (82.9)	4.29 (3.57)
Addicted: Very high/high tobacco dependence n = 37	161m (85.2m)	10.4 (8.47)	31,500 (11,900)	30,300 (11,500)	2,410 (1,520)	11,089,906 (4,183,164)	31 (77.5)	20.20 (7.69)	19 (47.5)	14 (35.0)	37 (92.5)	4.35 (3.40)
Total n = 75s	186m (150m)	7.47 (7.11)	26,000 (12,300)	25,000 (12,100)	2,030 (1,400)	18,243,906 (4,338,587)	60 (80.0)	16.6 (8.0)	35 (46.6)	23 (30.7)	66 (88.0)	4.32 (3.46)
P-value	0.2214	***0.002999	0.04198	*** p < 0.001	0.1474	0.04198^	0.7732	***.008496	1	0.4563	0.2894	0.1619

Note: Daily amount spent on waterpipe (VND) 37 out of 39 dual users provided waterpipe spending data; 39 out 75 were dual users. The factors that were significantly different by tobacco dependence were Percent of income spent on tobacco, Daily amount spent on tobacco (VND)*** and Daily amount spent on cigarettes (VND)***; ∞ while cigarette smoked per day also had a significant association with tobacco dependence, it is important to note that one of the six questions that make up the Fagerstrom scale which was used to define tobacco dependence includes this measure exactly “how many cigarettes a day do you smoke”. ^this variable, *Total spent on tobacco per year*, is constructed using the Daily amount spent on tobacco, therefore they have the same P-value

All three tobacco spending variables, the percent of annual income spent on tobacco, the daily cigarette expenditure, and the daily spending on tobacco of both cigarettes and waterpipe, were significantly positively associated with very high or high tobacco dependence (p = 0.01) in bivariate analysis (Table 2). In regression analysis, after controlling for potential confounders (age, income, years living with HIV, lives alone), the percentage of annual income spent on tobacco remained significant (p = 0.01) (Table 3). The regression results show that higher values of tobacco spending are independently associated with an increased likelihood of high tobacco dependence (very high/ high vs very low/low/medium). Therefore, individuals who have high/very high tobacco dependence spend on average 4.01% more on tobacco compared to those with very low/low/medium tobacco dependence. Regression analysis also shows that smokers with higher income spend more on tobacco (p = 0.01), and that those who live alone are more dependent on tobacco (p = 0.5).

**Table 3:** Factors associated with tobacco spending (percent of annual income) controlling for confounders (age, years living with HIV, living alone and income).

	Model 1	Model 2	Model 3	Model 4	Model 5
	coefficient/SE				
(Intercept)	4.11 ***	3.84 ***	3.84 ***	3.45 **	4.01 ***
	(1.08)	(1.08)	(1.09)	(1.08)	(1.03)
Tobacco dependence	6.31 ***	6.82 ***	6.81 ***	6.60 ***	5.86 ***
	(1.48)	(1.49)	(1.51)	(1.48)	(1.41)
Age		1.33	1.32	1.27	1.34
		(0.75)	(0.81)	(0.79)	(0.74)
Years Living with HIV			0.02	0.14	−0.31
			(0.79)	(0.78)	(0.74)
Lives Alone				4.70 *	3.18
				(2.34)	(2.25)
Income					−2.33 **
					(0.72)
N	75	75	75	75	75
AIC	495.52	494.30	496.30	494.10	485.46
BIC	502.47	503.57	507.89	508.01	501.69
Pseudo R2	0.20	0.23	0.23	0.27	0.37


All continuous predictors are mean-centered and scaled by 1 standard deviation. The outcome variable is in its original units. *** p < 0.001; ** p < 0.01; * p < 0.05.

### Robustness checks

Across all robustness checks, including both generalized and robust linear models, as well as the comparison between mixed-gender and male-only models, the strong, statistically significant positive association between tobacco spending as a percentage of annual income and tobacco dependence remained consistent. The negative association of tobacco spending with household income was not statistically significant in robust linear models.

### Interviews

#### Demographic characteristics of interview participants.

All participants were men aged between 36 and 57 years (mean SD age = 43.8 (5.3)). Half of them were dual users (n = 12), 42% (n = 10) had incomes ranging from 100 to 299 million VND (4,001 to 12,000 USD) per year, and 58% (n = 14) were married and had attained high school level education (Table 4).

**Table 4:** Characteristics of participants who completed in-depth interviews.

	n = 24
Variable	n (%) or *Mean (SD)*
Male	24 (100.0)
Age	*43.8 (5.3)*
Age of smoking initiation	*15.9 (4.1)*
Cigarettes smoked per day	*14.9 (8.9)*
Waterpipes smoked per day (dual users only, n = 12)	*9.3 (4.3)*
Married	14 (58.3)
Type of tobacco use	
Cigarette users	12 (50.0)
Dual users	12 (50.0)
Ever made a quit attempt (abstained from smoking for at least 24 hours)	14 (58.0)
Levels of education	
Primary school	4 (16.7)
Secondary school	6 (25.0)
High school	14 (58.3)
Household annual income in million VND (USD equivalent)	
300-499 m (12001–20000 USD)	1 (4.2)
100-299 m (4001–12000 USD)	10 (41.7)
50-99 m (2001–4000 USD)	9 (37.5)
10-49 m (400–2000 USD)	3 (12.5)
<9 m (<400 USD)	1 (4.2)

*Themes identified from interviews*

Three main themes emerged from our qualitative analysis:

(1) ***Perceived financial consequences of tobacco smoking***

Almost all participants, 23 out of 24, described tobacco products, i.e., both cigarettes and waterpipes as affordable.

Quote 1: “Tobacco is not too expensive. You see. It’s cheap. Generally, it’s in your affordability to do it, to earn and pay for it. The tobacco does not cost much.” – Cigarette smoker, 39 years old, divorced male.

Quote 2: “[…] it honestly doesn’t have much of an effect, only a few hundred thousand for cigarettes, so it doesn’t have any effect.” – Dual user, 54 years old, married male.

Five participants (21%) suggested that the financial burden differs for smokers who are employed or have a stable income, compared with smokers who are unemployed or do not have a stable income.

Quote 3: “[…] because we can still earn money, so... a few ten thousand[s] for a tobacco block is nothing. Later, when I am old... in several next few years, when I cannot work anymore, the finance might be affected.” – Cigarette smoker, 45 years old, married male.

Quote 4: “Honestly, regarding finance if I’m still working, it won’t affect anything. However, many old people are addicted. For example, people already in their 60s and 70s who still smoke a pack daily get affected. If you can’t work, then you obviously need to get money from your children for such things. You can’t get it from anyone else, so of course, it has effects.” – Dual smoker, 39 years old, single male.

Lack of stable employment was reported by one participant (4%) as negatively affecting his financial situation.

Quote 5: “Generally, as for farmers, smoking also affects the finances significantly. It is not that simple. There are ups and downs in my work, so it also affects a lot.” – Dual user, 40 years old, married male.

Saving money as a benefit of quitting smoking was acknowledged by one participant (4%).

Quote 6: “[…] generally I would also have more money to spend on other things which benefits me more [if I quit smoking]. I would have money for my children, to eat more, or to buy gifts, cookies, anything that could benefit my family.” – Cigarette smoker, 39 years old, divorced male.”

(2) ***Affordability of waterpipes compared to cigarettes***

Five dual users (21%) described switching to waterpipes to save money as waterpipes are much more affordable compared to cigarettes.

Quote 7: “[…] If I use cigarettes regularly, it is true that it will cost a lot of money, but I do not use it. But for waterpipe, it doesn’t matter much compared to my income or to my financial ability. Because one month I only use up about 60 thousand dong [about 2.50 USD]. So every day, it only costs about two thousand on average, it is not considered as... Talking about harm, it must be harmful, but talking about financial damage, it is not considered as much as a cigarette.” – Dual user, 48 years old, married male.

Quote 8: “I don’t think it affects me because every month, like I said, I only buy 100 grams of waterpipe for 60 thousand [dong] [2.5 USD] and I can smoke for a month, so it doesn’t affect me at all. Now it is said that buying a pack of cigarettes may cost forty or fifty thousand [dong] and, in a month, one pack a day, so people will spend a lot per month, so it affects their finances. It’s only that a pack of cigarettes that they buy can be as expensive as 100 grams of waterpipe that I buy and smoke for a month.” – Dual user, 44 years old, married male.

(3) *Financial motivation to quitting smoking ranged but was generally lacking.*

Saving money was not considered the main motivation for quitting in this sample. Instead, the prospect of improved health for themselves and their family was reported as the major motivation for quitting.

Quote 9: “I think… that [the main reason to quit] would be the [harmful] effects on the health of my family, myself. The most important point is that it would affect the health of my family. […] Secondly, it would be about finance.” – Dual smoker, 46 years old, married.

Quote 10: “It... means that if I quit tobacco then my health would be... better or... the finance could also be more stable.” – Cigarette smoker, 45 years old, married male.

Quote 11: “There are none [other reasons to quit], just mainly health. As for money, a few hundred thousand per month is no issue at all.” – Dual smoker, 54 years old, married male.

## Discussion

Our study examined tobacco spending among PWH who smoke and their perceptions about the affordability of tobacco products using both quantitative and qualitative data from PWH receiving care at OPCs in Hanoi, Viet Nam. Quantitatively, our sample spent on average 9.1 million VND per year on tobacco, which is the equivalent to 8% of the reported annual household income in this group. Moreover, we find a large proportion of participants (53%, n = 38) are addicted to nicotine. Qualitatively, we found respondents neither think the amount of money they spend on tobacco is significant, nor do they think this spending currently contributes to financial strain on their economic situation (e.g., displacing resources for their essential needs). Together, these findings suggest that though PWH who smoke are spending on tobacco, their perception is that the amount is not significant and not contributing to financial strain which could act as a motivation to quit. For example, the percentage of annual income spent on tobacco among PWH in Viet Nam is higher than that of other countries in the region (e.g., Cambodia, China), yet, tobacco-related spending is not a factor that motivates PWH who smoke in our study to quit [17–21]. Our findings may be partially explained by the atypical socioeconomic characteristics in our sample, which showed higher levels of employment, education, and income than those reported in previous studies of PWH who smoke and among the general population of tobacco users in Viet Nam. In light of the high level of tobacco addiction found in this sample, it is important to investigate how habits and ideas around tobacco spending are sometimes not aligned to further design appropriate smoking cessation interventions that target individual behaviors and to develop broader cessation policies targeting price, such as taxation of cigarettes.

### Socioeconomic characteristics, substitution of tobacco products, and general price

While we found the share of income spent on tobacco in our sample is high compared to other studies among PWH who smoke, overall, the perceptions of our participants do not reflect this data. Our participants generally perceived tobacco (both cigarettes and waterpipe) as affordable. These observations contrast with the existing literature, for example, a study in Kenya among PWH who smoke, where participants expressed concern about the financial burden associated with their tobacco use [49]. This divergence may be due to the stronger economic situation of our sample compared to previously studied HIV+ populations who use tobacco. For example, unemployment and food insecurity was low among our participants compared with previous studies, which largely represented economically vulnerable individuals like the unhoused or unemployed. Similarly, our participants may not view tobacco as cost prohibitive given that the majority of them, almost 90%, live with family in multigenerational households, where financial resources may be pooled. In such environments, the individual financial impact of spending on tobacco products may seem less significant because there are broader resources available from which to draw.

Notably, in this sample, a high usage of waterpipes is also reported, which provides a cheaper alternative to cigarettes. The few instances in which tobacco was described as inexpensive by participants were centered on comparisons between the cost of cigarettes and the cost of waterpipes. (Quote 8). For example, participants indicated switching between waterpipe and cigarette consumption to ease financial costs. Such substitution behavior may allow dual users to self-regulate their overall tobacco expenditure, potentially reducing their perception of tobacco-related expenses as a financial burden. Moreover, discounting tobacco-related expenditure as a motivation for quitting. Since waterpipe costs are not widely reported in tobacco use studies, it is difficult to understand its role in price-related substitution, which may occur among our population.

Lastly, tobacco may be seen as affordable by participants because objectively, the cost of purchasing cigarettes in Viet Nam is low, which is consistent with literature that describes the current pricing and taxation of cigarettes in the country as being greatly accessible compared to that of neighboring markets.

### Tobacco use in the Viet Nam context

Tobacco dependence is very high among this sample, as is spending on tobacco products. In our sample, PWH who smoke spend over three times more annually—approximately 2.7 million VND in 2020—compared to previous estimates among the general population in Viet Nam [23,24]. Additionally, we found that tobacco spending in our sample remained high across various demographics like age, education and income, which contrasts with patterns observed among the general population in HICs, where lower-income individuals spend more on tobacco compared to those with higher incomes. These findings suggest that the correlation between tobacco addiction and increased tobacco expenditure in this population, is higher than that of the general population in Viet Nam. This finding is consistent with other studies demonstrating high rates of smoking and addiction among PWH in other countries across Asia [17–21]. Specifically, there is high tobacco dependence among PWH and this strong dependence may contribute to a lack of motivation to quit.

There are several implications of this study. The data in our study suggests that the affordability of tobacco and tobacco related products in Viet Nam, undermines smoking cessation efforts by lessening the financial strain smoking places on consumers. Our study suggests that this correlation needs to be addressed in developing successful smoking cessation policies around tobacco costs. A U.S. study showed that a $1 excise tax hike on cigarettes led to a 9% decrease in affordability, showing that high taxation can be effective in reducing tobacco affordability [50, 51]. In Viet Nam, the WHO has recommended shifting to an excise tax model, which introduces the tax at the point of purchase and thus increases the sales price of tobacco products [15]. Similarly, two recent studies, one in Ha Noi and one in Da Nang, highlighted that the retail price of cigarettes remains low, and that the recent tax increase was insufficient to raise the current retail price significantly. This suggests a need to levy a specific and appropriate tax in order to substantially increase the price of cigarettes [52,53]. Another nationally representative study conducted across Viet Nam suggested that raising cigarette prices to at least 30,000 VND could decrease smoking rates [54]. The same study also found that increasing the visibility of health warning labels on cigarette packaging could also have a positive effect on smoking cessation rates. Our qualitative findings demonstrate that the prospect of improved health for our participants and their families was a major motivation for quitting. Thus, warning labels and health-centered messages that remind smokers of the health consequences of smoking could be particularly effective among PWH. Our observations also highlight the need for smoking cessation interventions tailored to PWH, which are currently not available at HIV OPCs in Viet Nam. Integrating smoking cessation counseling with routine HIV outpatient care offers a targeted approach to promote smoking cessation in this high-risk group.

To further investigate the relationship between tobacco expenditure and its financial impact on households where family members smoke, future research should focus beyond individual-level determinants of tobacco use and aim to explore socioeconomic factors and resources at the household level through longitudinal analysis. For example, data that accounts for income, access to financial support and tobacco use at the household level is important to provide a compressive picture of the burden of tobacco expenditure and how that burden may be spread across one household’s diverse financial resources. There is also a need to understand how the amount spent on cigarettes is connected to the actual price characteristics of specific tobacco purchases (e.g., price point, brand). Future research might employ economic methods to understand choices and prices. Additionally, further information is required on how pricing influences readiness to quit among PWH. Lastly, studies focused on PWH who smoke in Viet Nam must expand their geographic focus beyond Ha Noi and beyond clinical settings to capture variability in individual purchasing and spending, and prices of tobacco, which will vary by location.

### Limitations

Our study had several limitations. First, the quantitative sample size was dictated by time and budget constraints, resulting in a small sample that restricted a comprehensive examination of tobacco spending and its covariates. Second, the quantitative and qualitative samples are largely comprised of men due to the low prevalence of female smokers in Viet Nam, which limits the generalizability of our findings across genders. Third, our study did not capture data on cigarette types and brands, limiting our ability to assess cost variations and compare findings with other tax-specific studies that often make this distinction. Finally, although we included relevant confounding variables, unmeasured confounders may influence findings.

Despite these limitations, the independent qualitative and quantitative samples were drawn from the same narrow sampling frame and are demographically similar, enhancing coherence between datasets. Furthermore, our participants were PWH recruited from local OPCs where close to 90% of PWH receive care, thereby providing a strong reflection of the broader clinic-based population of PWH in Ha Noi.

## Conclusion

This mixed-methods study found PWH in Viet Nam who smoke spend a significant portion of their income on tobacco yet do not perceive this spending as burdensome. Quantitatively, participants showed high levels of tobacco addiction and reported that tobacco spending accounted for 7.47% of their annual income. Qualitatively, however, participants described smoking as affordable and revealed that tobacco spending did not serve as a motivation to quit smoking. Lowering tobacco consumption among PWH can lead to significant health improvements, including reducing premature deaths and the economic hardship of treating tobacco-related illnesses [55]. Our results show that current tobacco prices do not influence quitting behavior and highlight a need for stronger price-based interventions. Thus, in Viet Nam, priority should be given to creating taxation policies that lower the affordability of tobacco to reduce consumption, alongside integrating targeted smoking cessation interventions for PWH into routine HIV care.

## Supporting information

S1 Inclusivity-in-global-research-questionnaire. (DOCX)
